# Word-timestamped transcripts of two spoken narrative recall functional neuroimaging datasets

**DOI:** 10.1016/j.dib.2023.109490

**Published:** 2023-08-09

**Authors:** Savannah J. Born, Kathy Shi, Haemy Lee Masson, Hongmi Lee, Yoonjung Lee, Janice Chen

**Affiliations:** a Department of Psychological and Brain Sciences, Johns Hopkins University, Baltimore, Maryland, United States; b Department of Psychological & Brain Sciences, Washington University in St. Louis, St. Louis, MO, United States; c Department of Psychology, Durham University, Durham, United Kingdom; d Department of Psychological Sciences, Purdue University, West Lafayette, IN, United States

**Keywords:** Recollection, Speech production, Memory, Language, Naturalistic, fMRI

## Abstract

After watching audiovisual movies, human participants produced spoken narrative recollections during functional magnetic resonance imaging (fMRI); presented here are word-level timestamps of their speech, temporally aligned to the publicly shared fMRI data. For the “FilmFestival” dataset, twenty participants watched ten short audiovisual movies, approximately 2-8 minutes each. For the “Sherlock” dataset, seventeen participants watched the first half of the first episode of BBC's *Sherlock* (48 minutes). After viewing, participants then verbally described what they remembered about the movies in their own words. Participants’ speech was recorded using an MR-compatible microphone. The audio recordings were transcribed, then timestamped by a forced aligner; missing timestamps were filled in manually by human transcriptionists referencing the audio recording. Each file contains the participant's recall word by word, onset of each word in seconds with 1/10^th^-second precision, and the corresponding fMRI volume number (TR). This dataset can be used to investigate topics such as naturalistic memory and language production.

Specifications Table

**Table:** 

Subject	Experimental and Cognitive Psychology
Specific subject area	Naturalistic memory encoding and recall
Type of data	Spreadsheet
How the data were acquired	Audio was recorded using an Optoacoustics FOMRI III MR-compatible microphone and the software Audacity. Speech was transcribed by human listeners. Most timestamps were acquired using the Gentle forced aligner [5] and missing timestamps were filled in manually.
Data format	Raw
Description of data collection	Participants were healthy adults aged 18-45 who were right-handed native English speakers with normal or corrected vision, no history of neurological or psychological disorders, and no MRI contraindications. While in an MRI scanner, participants watched movies then verbally recounted what they remembered from the movies in their own words.
Data source location	“FilmFestival” fMRI data was collected at: Institution: Princeton University City: Princeton, New Jersey Country: USA “Sherlock” fMRI data was collected at: Institution: Princeton University City: Princeton, New Jersey Country: USA
Data accessibility	“Sherlock: Word Timestamps” and “FilmFestival: Word Timestamps” data are posted at https://zenodo.org/badge/latestdoi/630112473 "Sherlock" fMRI data are posted at two different repositories, in different formats; the “recall” portions of the datasets are not temporally aligned with each other. “Sherlock” fMRI data (Raw BIDS format): Repository name: OpenNeuro Data identification number: 10.18112/openneuro.ds001132.v1.0.0 Direct URL to data: https://openneuro.org/datasets/ds001132/versions/1.0.0 “Sherlock” fMRI data (Preprocessed fMRI) Repository name: Princeton DataSpace Data identification number: 10.1101/035931 Direct URL to data: https://dataspace.princeton.edu/handle/88435/dsp01nz8062179 “FilmFestival” fMRI data (Raw BIDS format): Repository name: OpenNeuro Data identification number: 10.18112/openneuro.ds004042.v1.0.0 Direct URL to data: https://openneuro.org/datasets/ds004042/versions/1.0.0
Related research article	Chen, J., Leong, Y., Honey, C. et al. Shared memories reveal shared structure in neural activity across individuals, Nat. Neurosci. 20, 115–125 (2017). https://doi.org/10.1038/nn.4450

## 1 Value of the Data

• The word-level timestamps are matched to the volume numbers of fMRI data collected as the participants spoke, therefore enabling time-resolved analyses of brain activity during speech.
• Scientists interested in questions about language can use these data to study brain activity during extended natural speech.
• Scientists interested in questions about memory can use these data to study brain activity during spontaneous spoken recollection.
• The speech was recorded as participants recalled an array of movies with widely varied content, providing opportunities for analyses of memories of stimulus features such as emotionality, social situations, music, and more.

## 2 Objective

This word-timestamps dataset supplements the “Sherlock” and “FilmFestival” fMRI datasets (results published in [1], [2], [3], [4]), facilitating future analysis. Participants’ speech has been analyzed to record the onset of each word, and aligned to the fMRI volume numbers (TRs). In prior publications, timestamps were only provided at the “scene” level, not at the word level.

## 3 Data Description

The “FilmFestival: Word Timestamps” dataset includes transcribed and timestamped verbal recollections of twenty human participants collected during a free spoken memory task following naturalistic movie-watching in an MRI scanner. The “Sherlock: Word Timestamps” dataset includes transcribed and timestamped verbal recollections of seventeen human participants. See Table 1, Table 2. Each excel spreadsheet (XLSX) contains participants’ verbal recollections timestamped at the word level and matched with the corresponding MRI volume number (TR). Each row of the XLSX file includes the transcription of the participant's utterance, the onset time of the utterance relative to the onset of the scanning run in seconds, the corresponding TR number, and the start time of the corresponding TR. See Table 3, Table 4.

**Table 1:** A list of the files from the “Sherlock: Word Timestamps” dataset.

Sherlock: Word Timestamps	Description	Age (years)	Sex (M/F)
NN01_JEX_181931_final	Timestamps corresponding to Sherlock_Princeton_Dataspace_fMRI_subject_01 and to OpenNeuro_fMRI_subject_01	20	M
NN02_AHQ_081013_final	Timestamps corresponding to Sherlock_Princeton_Dataspace_fMRI_subject_02 and to OpenNeuro_fMRI_subject_02	26	M
NN03_ANT_202231_final	Timestamps corresponding to Sherlock_Princeton_Dataspace_fMRI_subject_03 and to OpenNeuro_fMRI_subject_03	19	F
NN04_ATE_163931_final	Timestamps corresponding to Sherlock_Princeton_Dataspace_fMRI_subject_04 and to OpenNeuro_fMRI_subject_04	19	M
NN05_MUN_151631_final	Timestamps corresponding to Sherlock_Princeton_Dataspace_fMRI_subject_05	22	M
NN06_SQT_213031_final	Timestamps corresponding to Sherlock_Princeton_Dataspace_fMRI_subject_06 and to OpenNeuro_fMRI_subject_05	22	M
NN07_SKS_213031_final	Timestamps corresponding to Sherlock_Princeton_Dataspace_fMRI_subject_07 and to OpenNeuro_fMRI_subject_06	23	M
NN08_OLZ_224031_final	Timestamps corresponding to Sherlock_Princeton_Dataspace_fMRI_subject_08 and to OpenNeuro_fMRI_subject_07	22	F
NN09_NFO_231731_final	Timestamps corresponding to Sherlock_Princeton_Dataspace_fMRI_subject_09 and to OpenNeuro_fMRI_subject_08	19	M
NN10_NMM_233031_final	Timestamps corresponding to Sherlock_Princeton_Dataspace_fMRI_subject_10 and to OpenNeuro_fMRI_subject_09	21	M
NN11_NMA_233131_final	Timestamps corresponding to Sherlock_Princeton_Dataspace_fMRI_subject_11 and to OpenNeuro_fMRI_subject_10	20	F
NN12_DED_241531_final	Timestamps corresponding to Sherlock_Princeton_Dataspace_fMRI_subject_12 and to OpenNeuro_fMRI_subject_11	18	F
NN13_DII_241731_final	Timestamps corresponding to Sherlock_Princeton_Dataspace_fMRI_subject_13 and to OpenNeuro_fMRI_subject_12	20	F
NN14_DSN_242031_final	Timestamps corresponding to Sherlock_Princeton_Dataspace_fMRI_subject_14 and to OpenNeuro_fMRI_subject_13	21	M
NN15_DBF_242231_final	Timestamps corresponding to Sherlock_Princeton_Dataspace_fMRI_subject_15 and to OpenNeuro_fMRI_subject_14	22	M
NN16_DBN_242231_final	Timestamps corresponding to Sherlock_Princeton_Dataspace_fMRI_subject_16 and to OpenNeuro_fMRI_subject_15	21	F
NN17_DBE_242331_final	Timestamps corresponding to Sherlock_Princeton_Dataspace_fMRI_subject_17 and to OpenNeuro_fMRI_subject_16	19	F

**Table 2:** A list of files from the “FilmFestival: Word Timestamps” dataset. While there are 20 participants, there are 25 files because some participants’ spoken recall was split into two recordings due to their length. These subjects are JNB_194233, JNO_194133, ONH_224233, SBS_213433, and SKG_214133.

FilmFestival: Word Timestamps	Description	Age (years)	Sex (M/F)
ANE_164234_recallA_final	Timestamps corresponding to Filmfest_OpenNeuro_fMRI_subject_01	30	F
FHK_144134_recallA_final	Timestamps corresponding to Filmfest_OpenNeuro_fMRI_subject_02	22	F
FKB_143634_recallA_final	Timestamps corresponding to Filmfest_OpenNeuro_fMRI_subject_03	21	F
FKC_142334_recallA_final	Timestamps corresponding to Filmfest_OpenNeuro_fMRI_subject_04	25	M
FUL_143434_recallA_final	Timestamps corresponding to Filmfest_OpenNeuro_fMRI_subject_05	20	M
JLW_182834_recallA_final	Timestamps corresponding to Filmfest_OpenNeuro_fMRI_subject_06	22	M
JNB_193933_recallA_final	Timestamps corresponding to Filmfest_OpenNeuro_fMRI_subject_07 (note that a few words were spoken after the end of the fMRI scan)	28	F
JNB_194233_recallA1_final	Timestamps corresponding to Filmfest_OpenNeuro_fMRI_subject_08_recall1 (note that a few words were spoken after the end of the fMRI scan)	33	M
JNB_194233_recallA2_final	Timestamps corresponding to Filmfest_OpenNeuro_fMRI_subject_08_recall2		
JNN_193733_recallA_final	Timestamps corresponding to Filmfest_OpenNeuro_fMRI_subject_09	21	F
JNO_194133_recallA1_final	Timestamps corresponding to Filmfest_OpenNeuro_fMRI_subject_10_recall1	25	F
JNO_194133_recallA2_final	Timestamps corresponding to Filmfest_OpenNeuro_fMRI_subject_10_recall2		
JZQ_182934_recallA_final	Timestamps corresponding to Filmfest_OpenNeuro_fMRI_subject_11	23	F
MTU_151434_recallA_final	Timestamps corresponding to Filmfest_OpenNeuro_fMRI_subject_12	33	M
NOD_231733_recallA_final	Timestamps corresponding to Filmfest_OpenNeuro_fMRI_subject_13	31	F
NUX_231633_recallA_final	Timestamps corresponding to Filmfest_OpenNeuro_fMRI_subject_14	28	M
ONH_224233_recallA1_final	Timestamps corresponding to Filmfest_OpenNeuro_fMRI_subject_15_recall1 (note that a few words were spoken after the end of the fMRI scan)	25	M
ONH_224233_recallA2_final	Timestamps corresponding to Filmfest_OpenNeuro_fMRI_subject_15_recall2		
SBS_213433_recallA1_final	Timestamps corresponding to Filmfest_OpenNeuro_fMRI_subject_16_recall2. This audio file was of particularly low quality, and was very difficult to timestamp. Although the full transcript is included, timestamps for the first twelve minutes only are provided.	27	F
SBS_213433_recallA2_final	Timestamps corresponding to Filmfest_OpenNeuro_fMRI_subject_16_recall2		
SKB_214033_recallA_final	Timestamps corresponding to Filmfest_OpenNeuro_fMRI_subject_17	29	M
SKG_214133_recallA1_final	Timestamps corresponding to Filmfest_OpenNeuro_fMRI_subject_18_recall1 (note that a few words were spoken after the end of the fMRI scan)	27	F
SKG_214133_recallA2_final	Timestamps corresponding to Filmfest_OpenNeuro_fMRI_subject_18_recall2		
SLN_213333_recallA_final	Timestamps corresponding to Filmfest_OpenNeuro_fMRI_subject_19	28	M
SNE_213733_recallA_final	Timestamps corresponding to Filmfest_OpenNeuro_fMRI_subject_20	27	F

**Table 3:** An example of the format and columns included in each Sherlock timestamp file (example is from NN1_JEX_181931). Columns B-D give timing information for the words which are aligned to the Princeton Dataspace version of the fMRI data. Columns E-G give timing information aligned to the OpenNeuro version of the fMRI data.

A	B	C	D	E	F	G
Words	Princeton Dataspace Word Onset Time (sec)	Princeton Dataspace TR Number	Princeton Dataspace TR Onset (sec)	OpenNeuro Word Onset Time (sec)	OpenNeuro TR Number	OpenNeuro TR Onset (sec)
So	4.8	4	4.5	12.3	9	12
the	5.1	4	4.5	12.6	9	12

**Table 4:** An example of the format and columns included in each FilmFestival timestamp file (example is from ANE_164234_recallA). Columns B-D give timing information for the words which are aligned to the fMRI data posted on OpenNeuro. No other version of the fMRI data are publicly available at the time of this writing.

A	B	C	D
Words	Word Onset Time (sec)	TR Number	TR Onset Time (sec)
Okay	9.3	7	9
the	9.5	7	9

## 4 Experimental Design, Materials and Methods

While in an MRI scanner, participants watched audiovisual movies, then verbally recounted what they remembered from the movies in their own words. Participants' speech was recorded with an Optoacoustics FOMRI III MR-compatible microphone and the recording software Audacity. The fMRI data from two such experiments, as well as annotations of the movies, are posted publicly elsewhere (see Data Accessibility).

Human workers manually transcribed the audio recording of each subject's spoken recall and segmented each transcript into discrete sentences based on pauses in the speech and changes in topic. Timestamps for each word were first generated using the forced aligner Gentle [5]. Any missing timestamps were filled in manually by the same workers referencing the audio file using the software Audacity. The onset of the fMRI scan for each subject was then determined from the audio recording, and timestamps were adjusted to align with the fMRI volumes. The original audio recordings are not publicly shared due to privacy concerns.

The word timestamps are not adjusted to account for hemodynamic lag. In prior studies which used scene-level timestamps with the same primary fMRI data referenced here [1,2], timestamps were shifted by 3 TRs, relative to the brain data, in order to account for hemodynamic lag. For most analyses, we recommend applying a 3 TR shift for these word timestamps; for example, if the onset time given in this dataset for word X is t = 55 TRs, the corresponding BOLD response is considered to be at t = 58 TRs. Nonetheless, we supply the un-shifted word timestamps so that users can apply the parameters of their own choosing.

In the word timestamp spreadsheets, column A lists the transcribed speech, one word per row. Column B lists the onset time of each word in seconds (accurate to a tenth of a second), as identified by forced aligner or human judgment. Column C lists the onset time of each word in TRs, i.e., in which TR (brain volume number) the word began (sometimes words extend into the next TR). Column D lists the onset time (in seconds) of the TRs in Column C. If the word onset occurred exactly at the start boundary of a TR then the word was included as part of that TR. See Table 3, Table 4.

For Sherlock: Columns B-D give timing information for the words which are aligned to the Princeton Dataspace version of the fMRI data. Columns E-G give timing information aligned to the OpenNeuro version of the fMRI data. Note that subject numbers do not match between the two versions of the fMRI data: subject 5 is omitted in the OpenNeuro version; subjects 1-4 are the same between the two versions; subjects 6-17 in the Princeton Dataspace version correspond to subjects 5-16 in the OpenNeuro version. See Table 3.

For FilmFestival: Columns B-D give timing information for the words which are aligned to the fMRI data posted on OpenNeuro. No other versions of the fMRI data are publicly available at the time of this writing. See Table 4.

The word onset time is derived from the audio file. The TR number was calculated using the following formula in Excel: = CEILING(([word onset time] + 0.1) / 1.5, 1). The CEILING function rounds a number up away from zero to the desired multiple of significance. Since TRs are 1.5 seconds, dividing the word onset time by 1.5 and rounding up to the ones place would return the correct aligned TR. Rounding down would return the aligned TR minus one. 0.1 was added to the word onset time before division to account for instances where the word onset time is equal to a TR onset time. We considered words beginning at the same time as a TR as belonging to that TR, but the formula and CEILING function would return the previous TR. Therefore 0.1 was added to offset the word onset time so that when the time was divided by 1.5 the CEILING function would round up to the correct TR. See Table 5.

**Table 5:** Example of formula used to calculate the TR number from the word onset time. The formula we used is in Column B. We considered a word beginning at the same time as a TR as belonging to that TR. TR 2 begins at 1.5 seconds, so a word beginning at 1.5 seconds should be listed as TR 2. However, simply dividing the word onset time by 1.5 does not always return this, as shown in Column C. By adding 0.1 to 1.5 before dividing, instead of returning a whole value for the TR number (in this example, 1) that would not be rounded by the CEILING function, 1.1 is the result of the division which is rounded up by CEILING and correctly returns 2 as the TR number, as shown in Column B.

A	B	C
Word onset time (sec)	TR number calculated with formula =CEILING(([word onset time]+0.1)/1.5, 1)	TR number calculated with formula =CEILING(([word onset time])/1.5, 1)
1.4	1	1
1.5	2	1
1.6	2	2

The TR onset time was calculated in Excel using the formula: = ([TR number] * 1.5) - 1.5. Since the first TR, TR 1, begins at time zero, it is necessary to subtract 1.5 seconds when converting from TR number to TR onset time.

## Ethics Statements

Creation of the current word timestamps dataset did not involve any data collection with human subjects.

## CRediT authorship contribution statement

**Savannah J. Born:** Data curation, Investigation, Writing – original draft, Writing – review & editing. **Kathy Shi:** Software, Data curation, Writing – review & editing. **Haemy Lee Masson:** Data curation. **Hongmi Lee:** Data curation, Writing – review & editing. **Yoonjung Lee:** Data curation, Writing – review & editing. **Janice Chen:** Conceptualization, Methodology, Data curation, Investigation, Supervision, Funding acquisition, Writing – review & editing.

## Declaration of Competing Interest

The authors declare that they have no known competing financial interests or personal relationships that could have appeared to influence the work reported in this paper.

## Data Availability

Word Timestamped Transcripts (Original data) (Zenodo).
